# Biochemical deciphering of an Atypical methemoglobinemia

**DOI:** 10.1016/j.plabm.2026.e00527

**Published:** 2026-03-15

**Authors:** Antonin Lucbert, Hélène Caillon, Pierre-Olivier Bertho, Damien Masson, Manon Robert

**Affiliations:** Department of Biology, Laboratory of Clinical Biochemistry, Nantes Université, CHU Nantes, Nantes, France

**Keywords:** Methemoglobinemia, Glucose-6-phosphate dehydrogenase deficiency, Oxidation, Rasburicase

## Abstract

**Background:**

Methemoglobin is formed when the iron in hemoglobin is oxidized from the ferrous to the ferric state, preventing oxygen transport. This potentially life-threatening condition requires prompt diagnosis and treatment.

**Method:**

We report the case of a 30-year-old Senegalese man with newly diagnosed acute leukemia, admitted to the emergency department for suspected leukostasis syndrome. Induction chemotherapy and rasburicase were initiated. Unexpectedly, 24-h later, he developed acute hemolysis and a methemoglobinemia of 17%.

**Results:**

Investigation suggested rasburicase-induced methemoglobinemia. Rasburicase generates hydrogen peroxide during acid uric metabolism, normally neutralized by glutathione reductase, an enzyme that requires NADPH, which is produced via the pentose phosphate pathway. In this case, the presence of severe hemolytic anemia and marked susceptibility to oxidative stress prompted comprehensive laboratory investigations, including glucose-6-phosphate dehydrogenase deficiency testing, which returned positive. Supportive care, including monitoring, blood transfusions, and oxygen therapy, allowed normalization of methemoglobin levels within 48-h.

**Discussion:**

Methemoglobin can serve as a sentinel marker for the detection and monitoring of oxidative stress and related hematologic disorders. This case highlights the laboratory's role in identifying such abnormalities and the importance of clinician-laboratory awareness regarding rasburicase-induced methemoglobinemia. It also suggests the necessity of G6PD screening prior to rasburicase administration whenever possible.

## Highlights

• Rasburicase generates H_2_O_2_ normally neutralized by glutathione reductase and NADPH.
• Hemolytic anemia and methemoglobinemia can occur after rasburicase administration.
• Rasburicase-induced methemoglobinemia revealed G6PD deficiency.
• Suspected rasburicase-induced methemoglobinemia requires G6PD deficiency testing.
• If possible, test G6PD activity before rasburicase; if not, monitor methemoglobin.

## 1 Introduction

Hemoglobin is composed of a porphyrin heme structure, where iron is normally in the ferrous (Fe^2+^) state. Methemoglobinemia results from the oxidation of this ferrous iron to the ferric (Fe^3+^) state, forming methemoglobin, which is unable to bind oxygen effectively, leading to tissue hypoxemia [1,2]. Symptoms of methemoglobinemia include shortness of breath, cyanosis, headache, fatigue, dizziness, and confusion. If methemoglobin levels exceed 50%, patients may experience seizures, coma or death [2].

**Fig. 1:**
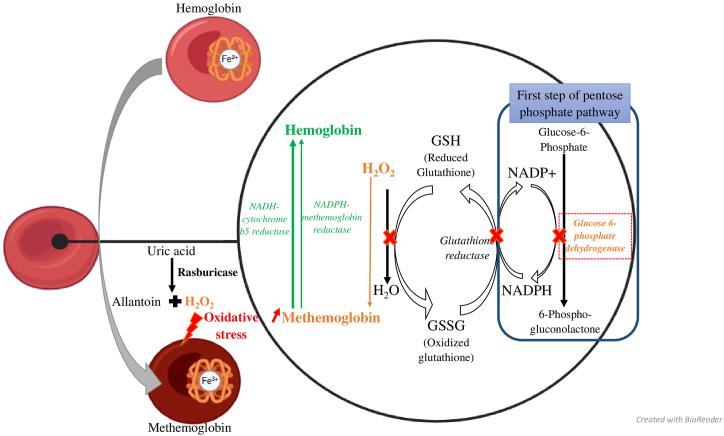
Mechanism of rasburicase-induced methemoglobinemia and the red-blood cell pathways involved in methemoglobin reduction. Methemoglobin results from the oxidation of hemoglobin iron. It is a reversible conversion in which, under normal conditions (in green), methemoglobin is reduced back to its ferrous state by enzymatic pathways. Two enzymes are involved: cytochrome *b*5 reductase, which is NADH-dependent, and NADPH-dependent methemoglobin reductase, which is mainly involved under conditions of oxidative stress. In this context, indirect endogenous protective mechanisms, including glutathione, also contribute. Uric acid is converted by the action of rasburicase into allantoin and hydrogen peroxide (H_2_O_2_). Hydrogen peroxide, shown in orange in the figure, is a reactive oxygen species responsible for oxidative stress. Under normal conditions, H_2_O_2_ is reduced to H_2_O by reduced glutathione. Reduced glutathione is regenerated by glutathione reductase from oxidized glutathione in an NADPH-dependent manner. In cases of G6PD deficiency, less NADPH is produced via the first step of the pentose phosphate pathway. As indicated by the red cross, this results in impaired regeneration of reduced glutathione, failure to detoxify H_2_O_2_, increased oxidative stress, iron oxidation, and subsequent methemoglobin formation.

Under normal conditions, methemoglobin accounts for about 1% of total hemoglobin due to the presence of endogenous reduction systems (Fig. 1) [2]. Methemoglobinemia is therefore a reversible form of dyshemoglobinemia, as enzymatic pathways enable the reduction of iron back to its ferrous state, primarily through the action of two enzymes represented in green in Fig. 1 [2]. Reduction of methemoglobin is primarily mediated by methemoglobin reductase I, also known as cytochrome *b*5 reductase, which is NADH- dependent. A second enzyme, NADPH-dependent methemoglobin reductase, also referred to as methemoglobin reductase II, plays a minor role under physiological conditions but contributes mainly in the presence of oxidative stress [2,3]. Indirect endogenous protective mechanisms against oxidative stress include sulfotransferase enzymes, ascorbic acid, and glutathione which is also represented in Fig. 1 [2].

Methemoglobinemia can result from a variety of causes, which may complicate its recognition and management, as physicians may not be familiar with all potential etiologies [2].

Methemoglobinemia is more frequently acquired, with the most common cause being ingestion or skin exposure to oxidizing agents. These include drugs such as sulfonamides, antimalarials and local anesthetics (e.g. Prilocaïne) [2,4] as well as substances derived from industrial chemical products (polyphenols, hydrazines, aniline, nitrobenzene). Other oxidizing agents, like chlorates and nitrates, which can be introduced through the diet (fertilizers, preservatives, well water) can also cause methemoglobinemia, as can the misuse of nitrate derivatives like poppers [5,6]. Oxidizing agents either react directly with hemoglobin to form methemoglobin or act indirectly by reducing oxygen to reactive oxygen species (free radical O_2_^−^, or water to hydrogen peroxide (H_2_O_2_)) responsible of hemoglobin oxidation. Rarely, methemoglobinemia has also been associated with food protein-induced enterocolitis syndrome (FPIES), in which intestinal inflammation leads to increased local nitrite production [7].

Less frequently, methemoglobinemia is hereditary due to congenital enzymatic deficiencies involving either cytochrome *b*5 reductase, which is inherited in an autosomal recessive manner, or NADPH- methemoglobin reductase II (rarer) [2,5]. Hemoglobin M disease, caused by mutations in the globin chains and inherited in an autosomal dominant manner, can also be responsible. This condition stabilizes iron in the ferric state, preventing its reduction and leading to persistent methemoglobin formation [2,8].

## 2 Clinical history

Here, we report the case of a 30-year-old Senegalese man with newly diagnosed acute myeloid leukemia, identified due to persistent deterioration of general condition and a fever ongoing for one week. He had no significant medical history. He developed respiratory symptoms, raising suspicion of leukostasis syndrome, which required admission to the intensive care unit. Initial biochemical analysis summarized at Table 1 revealed elevations of both LDH (963 IU/L) and uric acid (487 μmol/L). Induction chemotherapy (cytarabine and daunorubicin) was initiated, along with rasburicase to prevent tumor lysis syndrome. This resulting in a rapid decrease in uric acid levels. 24-h later, blood gas analysis unexpectedly revealed 17% methemoglobinemia, despite the lack of cyanosis symptoms. 48-h later, LDH rose sharply to 4008 IU/L, acid uric decreased (<12 μmol/L), hemoglobin dropped to 3.6 g/dL, and haptoglobin was undetectable (Table 1).

**Table 1:** Patient results before and 48-h after rasburicase administration.

Analyte	Results		Reference interval
	Prior to rasburicase administration	48-h after rasburicase administration	
**Hemoglobin**	6.6 g/dL	3.6 g/dL	13.5-17.5 g/dL
**Total calcium**	2.21 mmol/L	2.24 mmol/L	2.25-2.50 mmol/L
**Phosphate**	1.46 mmol/L	0.84 mmol/L	0.81-1.45 mmol/L
**Creatinine**	79 μmol/L	106 μmol/L	62-106 μmol/L
**Uric acid**	487 μmol/L	<12 μmol/L	200-415 μmol/L
**Lactate dehydrogenase**	963 IU/L	4008 IU/L	135-225 IU/L
**Haptoglobin**	NP	<0.1 g/L	0.6-1.6 g/L

NP: Not performed.

### 2.1 Laboratory role in diagnosis, mechanistic insights and patient management

The etiology of this methemoglobinemia was then investigated. Hemoglobin electrophoresis was performed, notably to rule out Hemoglobin M disease, and the electrophoretic pattern was normal. The patient denied ingestion of oxidizing agents such as poppers. Rasburicase was therefore considered one of the most plausible causative agents. Indeed, rasburicase produces hydrogen peroxide while converting uric acid to allantoin. This oxidizing agent is normally neutralized by glutathione reductase, an enzyme that requires NADPH, which is produced via the first step of the pentose-phosphate pathway represented in Fig. 1, likely defective in this case [9,10]. Additionally, the presence of severe hemolytic anemia, characterized by high levels of LDH, undetectable haptoglobin and a hemoglobin drop to 3.6g/dL (Table 1), raised suspicion of glucose-6-phosphate dehydrogenase (G6PD) deficiency. This was subsequently confirmed by a reduced enzyme activity of 5.9 IU/g of hemoglobin (reference range: 11–17 IU/g of hemoglobin). The pathophysiological mechanism involve in this case is illustrated in Fig. 1: due to G6PD deficiency, less NADPH is produced via the pentose-phosphate pathway, impairing the regeneration of reduced glutathione by glutathione reductase. As a result, detoxification of hydrogen peroxide is compromised, leading to oxidative stress, iron oxidation, and methemoglobin formation [9,11]. For this patient, methylene blue was contraindicated and rasburicase stopped and replaced with allopurinol. He received multiple units of packed red blood cells, and early oro-tracheal intubation was initiated, allowing methemoglobinemia levels to decrease within 48-h (dropping below 10% after 24-h).

## 3 Discussion

This case underscores the diagnostic approach to methemoglobinemia and emphasizes the cellular signaling pathways responsible for maintaining iron in its ferrous state. The concurrent presentation of severe hemolytic anemia and methemoglobinemia following rasburicase administration should alert clinicians and laboratory professionals to the possibility of G6PD deficiency, even in patients without any prior medical history.

The rarity of this situation is reflected by others reports of rasburicase-induced methemoglobinemia [10,[12], [13], [14]], with the first occurrence case described in 2005 [14]. A review conducted by Hammami and colleagues identified only 43 cases worldwide as of September 2024 [10]. Most affected patients had been previously diagnosed with lymphoma or leukemia like in our case [14]. A median time to symptom onset of 24-h and a median methemoglobin level of 10% were reported, comparable to the patient presented here [10]. Similar to our case, G6PD deficiency was frequently diagnosed only after rasburicase administration. Four deaths have been reported, with a median time to death of 8 days. Treatment approaches included blood transfusion, discontinuation of rasburicase, and ascorbic acid administration [10]. Patients were usually managed with supportive care and red blood cells transfusion [12]. Among patients who recovered, the median time to clinical improvement was 4.5 days [10], one study reported long time recovery of 25 days [15]. Interestingly, no clear correlation has been established between rasburicase dose and levels of methemoglobin or hemoglobin [13]. Importantly, rasburicase-induced methemoglobinemia is not limited to specific ethnic groups; Caucasian patients can also be exceptionally affected, as illustrated by Raru and collaborators [13]. This case highlights the need for vigilant monitoring after rasburicase administration, even though complications like methemoglobinemia and hemolysis are rare, to enable early intervention. Furthermore, it raises an important clinical issue regarding the management of methemoglobinemia in G6PD-deficient patients, particularly the controversial use of methylene blue in this population [2]. It may be advisable to systematically screen for G6PD deficiency through laboratory testing before administering rasburicase, when possible, as is done with dapsone.

Methemoglobinemia is a life-threatening condition that must be promptly recognized and treated [2]. The first step in management is the removal of the causative agent. Other treatments measures include the administration of antidotal therapy (methylene blue), blood transfusion, oxygen therapy and close monitoring [2,14]. Methylene blue can significantly reduce methemoglobin levels within an hour, and is recommended in cases of methemoglobinemia exceeding 20%, when symptoms or comorbid conditions are present [2,5]. Laboratory vigilance remains warranted, as technical difficulties in assessing methemoglobin levels by co-oximetry have been reported following methylene blue administration [16]. Paradoxically, methylene blue is an oxidizing agent that is enzymatically reduced to leucomethylene blue, a reducing agent, by NADPH-dependent methemoglobin reductase. Leucomethylene blue then restores hemoglobin function by reducing ferric iron in methemoglobin to its functional ferrous state via electron transfer [2]. It is important to note that methylene blue should be avoided in patients with G6PD deficiency. These individuals produce insufficient amounts of NADPH, leading to inadequate reduction of methylene blue to leucomethylene blue. As a result, methylene blue accumulate as an oxidizing agent, potentially exacerbating methemoglobinemia and hemolytic anemia [2]. As in our case, most reports in the literature indicate that oxygen supplementation and blood transfusion are often sufficient to manage methemoglobinemia [14]. Exchange transfusion has been reported as a potentially successful therapeutic option [10]. Less invasive alternatives have also been proposed, such as N-acetylcysteine, which is believed to act both as a precursor for glutathione synthesis and as an electron donor [2]; and ascorbic acid, a reducing agent involved in various oxidation-reduction reactions [14]. Additionally, dextrose administration has been suggested, as it provides a source of NADH and NADPH in red blood cells [14].

## 4 Conclusion

This case illustrates that methemoglobin can serve as a sentinel marker in the detection and monitoring of oxidative stress and related hematologic disorders, as shown in Fig. 1. In this instance, the occurrence of methemoglobinemia at 17% in a patient hospitalized for acute leukemia prompted an investigation into the underlying etiology. Rasburicase was considered the most likely cause. A potential impairment of oxidative stress defense mechanisms was considered. Given the presence of acute hemolysis, the patient's ethnic origin, G6PD deficiency was suspected and confirmed. It underscores the crucial role of the laboratory in identifying such abnormalities, highlights the need to consider methemoglobinemia as a potential adverse effect of rasburicase and suggests the necessity of G6PD screening prior to rasburicase administration; if screening is not feasible, close monitoring of methemoglobin levels is recommended. Recognition of these findings directly influences patient management and therapeutic decisions; in this case, it led to discontinuation of rasburicase and initiation of supportive care (transfusion and intubation). Notably, in cases of more severe methemoglobinemia, methylene blue may not be advisable in patients with G6PD deficiency.

## CRediT authorship contribution statement

**Antonin Lucbert:** Writing – original draft, Validation, Conceptualization. **Hélène Caillon:** Writing – review & editing, Validation, Supervision. **Pierre-Olivier Bertho:** Validation. **Damien Masson:** Writing – review & editing, Validation, Supervision. **Manon Robert:** Writing – review & editing, Validation, Supervision.

## Funding

None.

## Declaration of competing interest

The author declare no conflict of interest.

## Data Availability

No data was used for the research described in the article.

## References

[1] Gao H., Basri R., Tran M.H. (2022). Acquired methemoglobinemia: a systematic review of reported cases. Transfus. Apher. Sci.: Official Journal of the World Apheresis Association: Official Journal of the European Society for Haemapheresis.

[2] Wright R.O., Lewander W.J., Woolf A.D. (1999). Methemoglobinemia: etiology, pharmacology, and clinical management. Ann. Emerg. Med..

[3] Umbreit J. (2007). Methemoglobin--it’s not just blue: a concise review. Am. J. Hematol..

[4] Kumar A.R., Dunn N., Naqvi M. (1997). Methemoglobinemia associated with a prilocaine-lidocaine cream. Clin. Pediatr..

[5] Skold A., Cosco D.L., Klein R. (2011). Methemoglobinemia: pathogenesis, diagnosis, and management. South. Med. J..

[6] Lefevre T., Nuzzo A., Mégarbane B. (2018). Poppers-induced life-threatening methemoglobinemia. Am. J. Respir. Crit. Care Med..

[7] Genere L., Pecciarini N., Peretti N. (2017). [food protein-induced enterocolitis syndrome: a case report of diarrhea with hypovolemic shock and methemoglobinemia]. Arch. Pediatr.: Organe Officiel De La Societe Francaise De Pediatrie.

[8] Pulsinelli P.D., Perutz M.F., Nagel R.L. (1973). Structure of hemoglobin M Boston, a variant with a five-coordinated ferric heme. Proc. Natl. Acad. Sci. U. S. A..

[9] Luzzatto L., Nannelli C., Notaro R. (2016). Glucose-6-Phosphate dehydrogenase deficiency. Hematol. Oncol. Clin. N. Am..

[10] Hammami M.B., Qasim A., Thakur R. (2024). Rasburicase-induced hemolytic anemia and methemoglobinemia: a systematic review of current reports. Ann. Hematol..

[11] Cappellini M.D., Fiorelli G. (2008). Glucose-6-phosphate dehydrogenase deficiency. Lancet.

[12] Ibrahim U., Saqib A., Mohammad F. (2018). Rasburicase-induced methemoglobinemia: the eyes do not see what the mind does not know. J. Oncol. Pharm. Pract.: Official Publication of the International Society of Oncology Pharmacy Practitioners.

[13] Raru Y., Abouzid M., Parsons J. (2019). Rasburicase induced severe hemolysis and methemoglobinemia in a Caucasian patient complicated by acute renal failure and ARDS. Respiratory Medicine Case Reports.

[14] Vidhyashree B.H., Zuber M., Taj S. (2022). Rasburicase induced methemoglobinemia: a systematic review of descriptive studies. J. Oncol. Pharm. Pract.: Official Publication of the International Society of Oncology Pharmacy Practitioners.

[15] Sherwood G.B., Paschal R.D., Adamski J. (2016). Rasburicase-induced methemoglobinemia: case report, literature review, and proposed treatment algorithm. Clin. Case Rep..

[16] Haymond S., Cariappa R., Eby C.S. (2005). Laboratory assessment of oxygenation in methemoglobinemia. Clin. Chem..

